# Comparative Analysis of AbaR-Type Genomic Islands Reveals Distinct Patterns of Genetic Features in Elements with Different Backbones

**DOI:** 10.1128/mSphere.00349-20

**Published:** 2020-05-27

**Authors:** Dexi Bi, Jiayi Zheng, Ruting Xie, Yin Zhu, Rong Wei, Hong-Yu Ou, Qing Wei, Huanlong Qin

**Affiliations:** a Department of Pathology, Shanghai Tenth People’s Hospital, Tongji University School of Medicine, Shanghai, China; b Department of Gastrointestinal Surgery, Shanghai Tenth People’s Hospital, Tongji University School of Medicine, Shanghai, China; c State Key Laboratory of Microbial Metabolism, Shanghai-Islamabad-Belgrade Joint Innovation Center on Antibacterial Resistances, and School of Life Sciences and Biotechnology, Shanghai Jiao Tong University, Shanghai, China; Antimicrobial Development Specialists, LLC

**Keywords:** antimicrobial resistance, evolution, genomic island

## Abstract

AbaR-type genomic islands (AbaRs) are prevalent and associated with multiple antimicrobial resistance in Acinetobacter baumannii. AbaRs feature varied structural configurations involving different but closely related backbones with acquisition of diverse mobile genetic elements (MGEs) and antimicrobial resistance genes. This study aimed to understand the structural modulation patterns of AbaRs. A total of 442 intact AbaRs, including nonresistance but closely related islands, were mapped to backbones Tn*6019*, Tn*6022*, Tn*6172*/Tn*6173*, and AbGRI1-0 followed by alien sequence characterization. Genetic configurations were then examined and compared. The AbaRs fall into 53 genetic configurations, among which 26 were novel, including one Tn*6019*-type, nine Tn*6022*-type, three Tn*6172*/Tn*6173*-type, nine AbGRI1-type, and four new transposons that could not be mapped to the known backbones. The newly identified genetic configurations involved insertions of novel MGEs like IS*Acsp2*, IS*Aba42*, IS*Aba17*, and IS*Aba10*, novel structural modulations driven by known MGEs such as IS*CR2*, Tn*2006*, and even another AbaR, and different backbone deletions. Recombination events in AbGRI1-type elements were also examined by identifying hybrid sequences from different backbones. Moreover, we found that the content and context features of AbaRs including the profiles of the MGEs driving the plasticity of these elements and the consequently acquired antimicrobial resistance genes, insertion sites, and clonal distribution displayed backbone-specific patterns. This study provides a comprehensive view of the genetic features of AbaRs.

**IMPORTANCE** AbaR-type genomic islands (AbaRs) are well-known elements that can cause antimicrobial resistance in Acinetobacter baumannii. These elements contain diverse and complex genetic configurations involving different but related backbones with acquisition of diverse mobile genetic elements and antimicrobial resistance genes. Understanding their structural diversity is far from complete. In this study, we performed a large-scale comparative analysis of AbaRs, including nonresistance but closely related islands. Our findings offered a comprehensive and interesting view of their genetic features, which allowed us to correlate the structural modulation signatures, antimicrobial resistance patterns, insertion loci, as well as host clonal distribution of these elements to backbone types. This study provides insights into the evolution of these elements, explains the association between their antimicrobial resistance gene profiles and clonal distribution, and could facilitate establishment of a more proper nomenclature than the term “AbaR” that has been variously used.

## Abstract (precis)

AbaR-type genomic islands (AbaRs) are well-known elements that can cause antimicrobial resistance in Acinetobacter baumannii. These elements contain diverse and complex genetic configurations involving different but related backbones with acquisition of diverse mobile genetic elements and antimicrobial resistance genes. Understanding their structural diversity is far from complete. In this study, we performed a large-scale comparative analysis of AbaRs, including nonresistance but closely related islands. Our findings offered a comprehensive and interesting view of their genetic features, which allowed us to correlate the structural modulation signatures, antimicrobial resistance patterns, insertion loci, as well as host clonal distribution of these elements to backbone types. This study provides insights into the evolution of these elements, explains the association between their antimicrobial resistance gene profiles and clonal distribution, and could facilitate establishment of a more proper nomenclature than the term “AbaR” that has been variously used.

## INTRODUCTION

Multidrug-resistant Acinetobacter baumannii remains a major threat of nosocomial infection. AbaR-type genomic islands (AbaRs) are a class of important mobile genetic elements (MGEs) prevalent in A. baumannii and have been reported to carry multiple antimicrobial resistance genes (1–4).

AbaRs exhibit variable genetic structural features involving certain different but closely related backbones and diverse acquired transposons, insertion sequences (IS), and antimicrobial resistance genes based on these genetic elements (5–8). Tn*6019*, Tn*6022*, and Tn*6172*/Tn*6173* have been recognized as backbone elements (5, 8–10). Tn*6173* is a hypothetical transposon from which Tn*6172* is derived (8). These backbones share a 2.89-kb left-end conserved sequence (CS) and a 1.87-kb right-end CS spanning transposition genes (4). AbaRs commonly disrupt the chromosomal *comM* gene. Elements with different backbones are usually found in different epidemic clones. The well-documented AbaR3-type elements are seemingly confined to global clone 1 (GC1) (3). They contain a Tn*6019* backbone and are invariably associated with Tn*6018* or its compound elements containing plastic multiple antimicrobial resistance regions (MARRs) (3). The Tn*6022*-derived elements mostly are found in GC2 and sometimes bear the *bla*_OXA-23_ gene as seen in AbaR4 (9). A class of complex elements called AbGRI1-type islands have recently been identified in GC2, which are proposed to originate from a plasmid-borne ancestral form (AbGRI1-0) consisting of a Tn*6022*, a Tn*6172*, and a plasmid-borne fragment between the two transposons (termed “linker” here) as a transposable unit (8, 12). Our recent large-scale identification of AbaRs in A. baumannii genomes have also uncovered that they have diverse insertion sites and clonal lineage-specific antimicrobial resistance gene profiles (4). Although efforts have been made to study the evolution of AbaRs (6, 13–15), understanding of their structural diversity is far from complete.

The term “AbaR” was initially coined for A. baumannii resistance islands (1). It has also been used for highly homologous islands without resistance genes that were discovered later. All these elements displayed wide diversity, but they could be genetically mapped to certain backbones as mentioned above, regardless of whether they carried resistance genes. There is still a lack of consensus on the nomenclature of these elements. For simplicity, here we collectively called these islands “AbaRs” (4). To avoid confusion, it should be noted that the term was used only to refer to these genetically related islands and was not limited to islands with resistance genes.

In this study, exhaustive profiling and comparative analysis of the genetic features of 422 intact AbaRs with complete genetic information were performed, which allows us to correlate their backbone types with structural modulation signatures, antimicrobial resistance patterns, insertion loci, as well as clonal distribution.

## RESULTS

### Twenty-six novel AbaR genetic configurations were identified.

A total of 442 AbaRs were selected (see Data Set S1 in the supplemental material), among which 65 nonredundant elements were identified. They were further mapped to backbones Tn*6019* (5), Tn*6022* (9), Tn*6172* (10), Tn*6173* (8), and AbGRI1-0 (8), followed by comparative analysis. AbaRs were characterized into 53 genetic configurations (see Data Set S1 and Table S1 in the supplemental material). Three configurations were present as intact backbones, including Tn*6019*, Tn*6022*, and Tn*6172*, 46 were variants associated with the known backbones, while four (designated Tn*6661* to Tn*6664*) were new transposons that could not be mapped to any of the known backbones. Of note, 26 configurations were novel ones, including one Tn*6019*-derived, nine Tn*6022*-derived, three Tn*6172*/Tn*6173*-derived, nine AbGRI1-type, and four new transposons.

10.1128/mSphere.00349-20.5 DATA SET S1 Intact AbaRs analyzed in this study. Download Data Set S1, XLS file, 0.2 MB. Copyright © 2020 Bi et al. 2020 Bi et al. This content is distributed under the terms of the Creative Commons Attribution 4.0 International license.

10.1128/mSphere.00349-20.6 TABLE S1 Genetic configurations of AbaR-type genomic islands and their features. Download Table S1, DOCX file, 0.1 MB. Copyright © 2020 Bi et al. 2020 Bi et al. This content is distributed under the terms of the Creative Commons Attribution 4.0 International license.

### Comparative analysis of Tn*6019*-derived elements.

**FIG 1:**
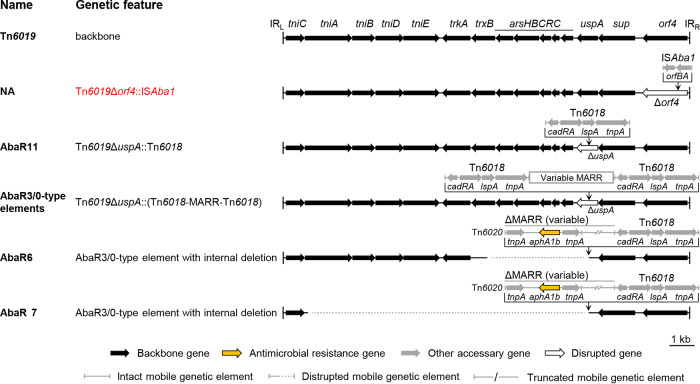
Comparison of Tn*6019*-derived genetic configurations. The Tn*6019* backbone is shown at the top of the figure. The backbone segments in the variants are placed in aligned positions. The names of the reported elements bearing the corresponding genetic configurations are given. NA, not available. The novel genetic configuration is shown in red. Dotted lines are used to connect sequences. Detailed genetic organizations of the variable multiple antimicrobial resistance regions (MARRs) or truncated MARRs for specific AbaR3/0-type elements are not shown. The schematic representations are drawn to scale, except for the MARRs.

Five configurations were found to be based on Tn*6019* (Fig. 1). Four of the configurations were associated with Tn*6018* or its compound elements that disrupted the *uspA* gene and have been widely seen and well documented (3). We found a novel configuration that had an IS*Aba1* disrupting *orf4*, but there was only one element bearing this configuration. Note that AbaRs containing the Tn*6018* compound elements were characterized as one configuration (type) here for simplicity despite the variable nature of the internal MARRs (3), unless there were subsequent deletions on the backbones.

### Comparative analysis of Tn*6022*-derived elements.

**FIG 2:**
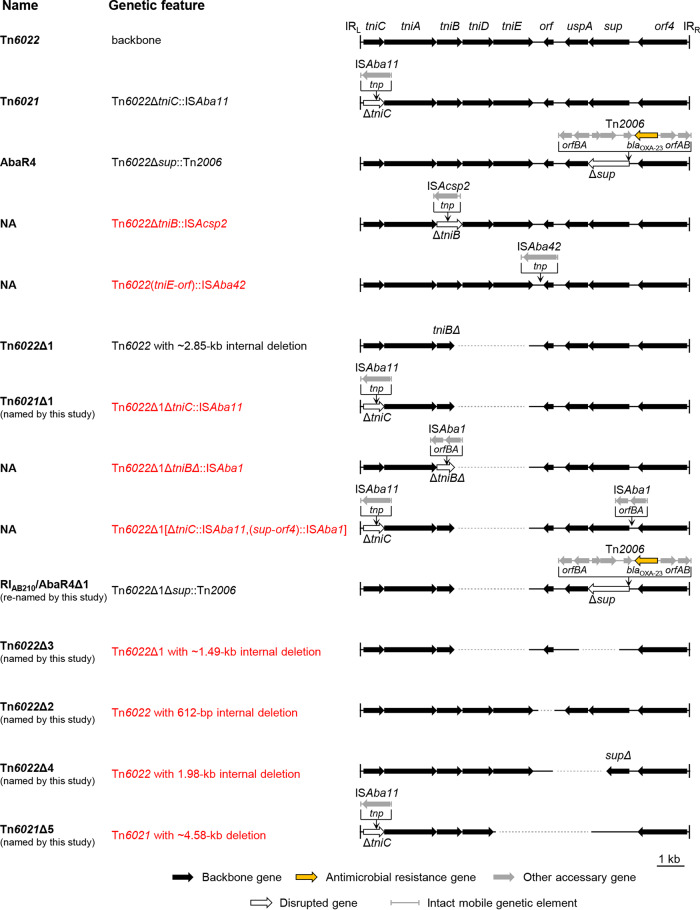
Comparison of Tn*6022*-derived genetic configurations. The Tn*6022* backbone is shown at the top of the figure. The backbone segments in the variants are placed in aligned positions. The names of the reported elements bearing the corresponding genetic configurations are given. NA, not available. The names given by this study are also shown. Novel genetic configurations are shown in red. Dotted lines are used to connect sequences. The schematic representations are drawn to scale.

Thirteen configurations were characterized as Tn*6022* derived (Fig. 2), with variations involving insertions of diverse MGEs and backbone deletions. The IS*Aba11* and Tn*2006* (IS*Aba1*-bounded composite transposon) were frequently seen and site specifically disrupted *tniC* and *sup*, respectively. Insertions of IS*Acsp2*, IS*Aba42*, and IS*Aba1* were newly found. IS*Acsp2* disrupted *tniB*, the IS*Aba42* inserted into the intergenic region between *tniE* and *orf*, while IS*Aba1* was seen at different sites. Four novel deletion forms of the backbone were found in addition to Tn*6022*Δ1 (2). The Tn*6022*Δ3 had an extra 1.49-kb deletion based on Tn*6022*Δ1, while in Tn*6022*Δ2, Tn*6022*Δ4, and Tn*6021*Δ5, different internal fragments (0.6 to 4.6 kb) were missing. Note that the deletion in Tn*6022*Δ2 likely involved 6-bp perfect duplicated target sequences (5′-AAATGC-3′), suggesting the missing fragment might be a novel IS.

### Comparative analysis of Tn*6172*/Tn*6173*-derived elements.

**FIG 3:**
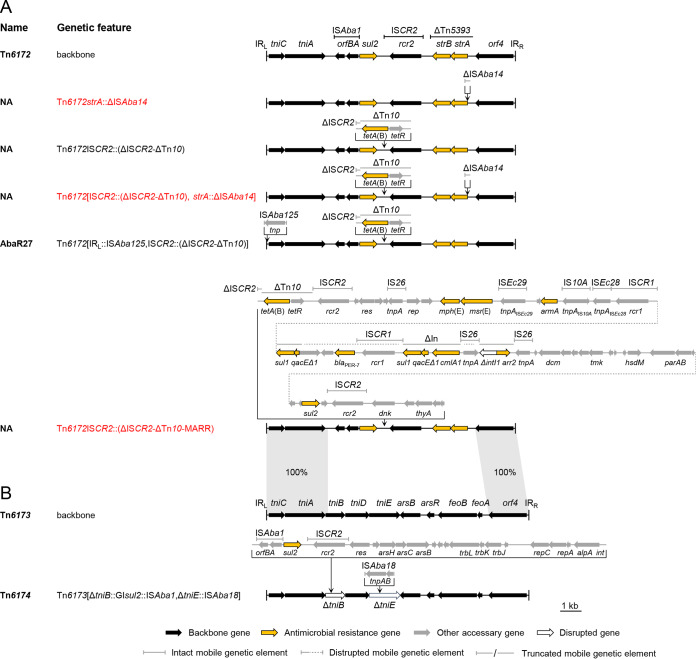
Comparison of Tn*6172*- or Tn*6173*-derived genetic configurations. (A) Tn*6172*-derived variants. (B) Tn*6173*-derived variant. The backbones are shown at the top of the panels. The backbone segments in the variants are placed in aligned positions. The names of the reported elements bearing the corresponding genetic configurations are given. NA, not available. Novel genetic configurations are shown in red. Dotted lines are used to connect sequences. Syntenic regions between Tn*6172* and Tn*6173* are connected by gray. The schematic representations are drawn to scale.

Six configurations were mapped to Tn*6172*/Tn*6173* (Fig. 3). A ΔIS*CR2*-ΔTn*10* fragment containing an *tet*(B) resistance gene (8) was frequently seen at the left end of the IS*CR2* of Tn*6172*. We newly found an addition of a truncated IS*Aba14* upstream of *strA* on Tn*6172* (10) or Tn*6172*IS*CR2*::(ΔIS*CR2*-ΔTn*10*) (8). We also found a configuration uncommon to this type, which contained a large fragment consisting of multiple antimicrobial resistance genes and diverse ISs. Interestingly, at the left end of the fragment lay a ΔIS*CR2*-ΔTn*10* structure as well, and the fragment shared the same insertion loci with ΔIS*CR2*-ΔTn*10*.

### Comparative analysis of AbGRI1-type elements.

**FIG 4:**
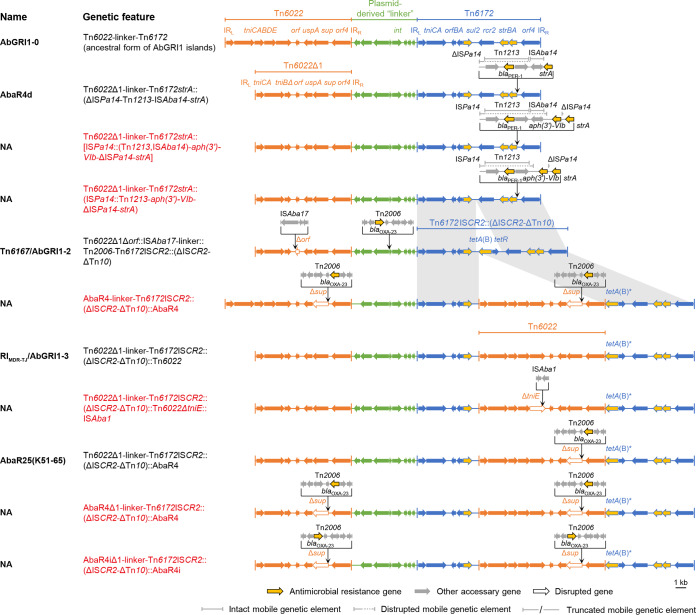
Genetic configurations of the variants with a typical AbGRI1 structure. In this study, the typical AbGRI1 structure was defined as an element containing an intact “Tn*6022*-linker-Tn*6172*” backbone. The proposed ancestral form (AbGRI1-0) of AbGRI1-type elements is shown above. The Tn*6022* or ΔTn*6022* part is shown in orange, the Tn*6172* part or its partial segments are shown in blue, while the linker region is shown in green. The backbone segments in the variants are placed in aligned positions or otherwise connected by gray. The syntenic regions between Tn*6172*IS*CR2*::(ΔIS*CR2*-ΔTn*10*) and its disrupted form are also connected by gray. The names of the reported elements bearing the corresponding genetic configurations are given. NA, not available. Novel genetic configurations are shown in red. The letter “i” in the names “AbaR4iΔ1” and “AbaR4i” indicates that the Tn*2006* transposon in AbaR4iΔ1 and AbaR4i are inverted compared to that in AbaR4Δ1 and AbaR4, respectively. The schematic representations are drawn to scale.

**FIG 5:**
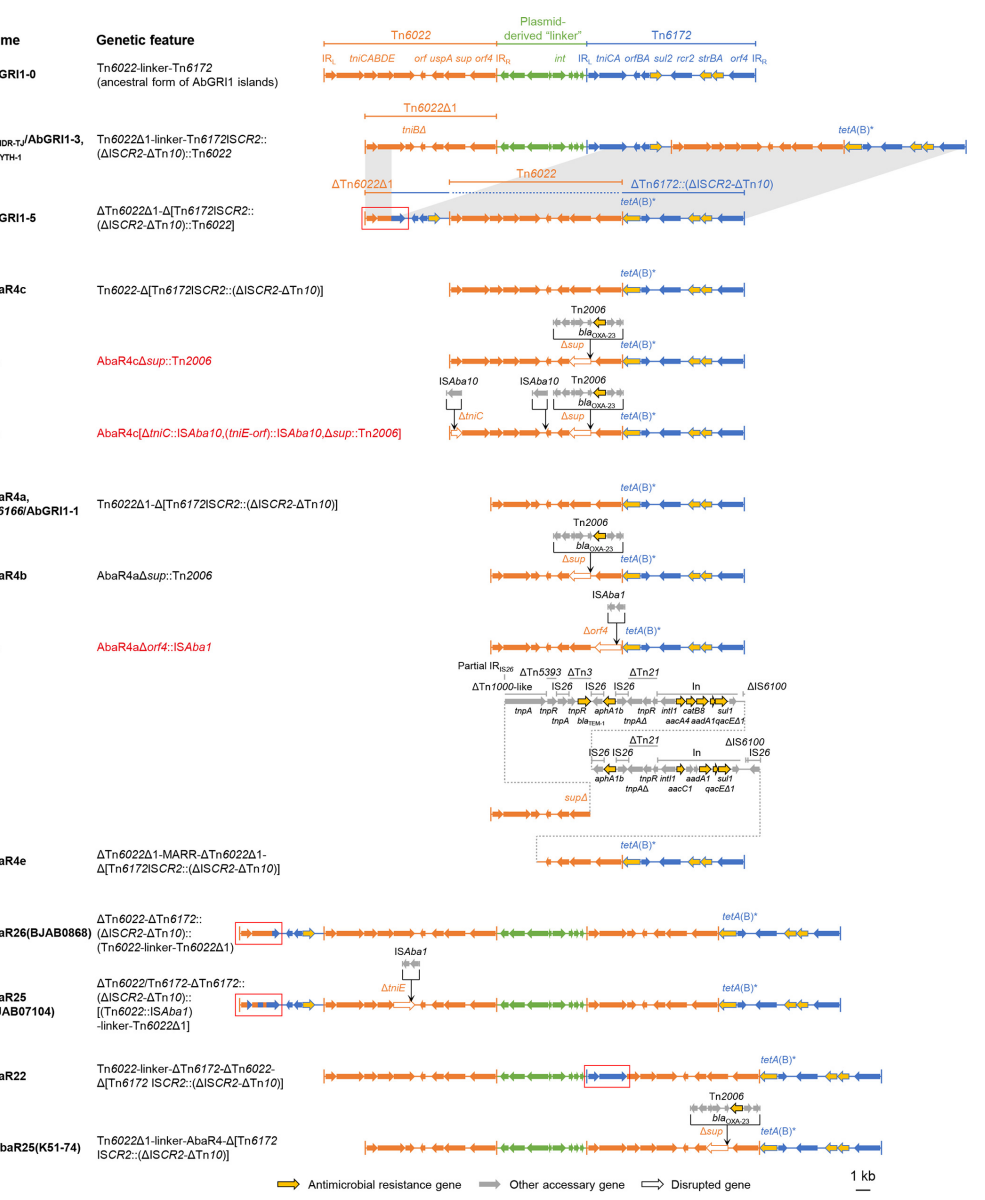
Genetic configurations of the AbGRI1-type variants that might have gone through recombination. The proposed ancestral form (AbGRI1-0) of AbGRI1-type elements is shown at the top of the figure. An example of a proposed recombination process is shown (see AbGRI1-3 and AbGRI1-5) (16). The syntenic regions with near 100% identity between AbGRI1-3 and AbGRI1-5 are connected by gray. The Tn*6022* or ΔTn*6022* part is shown in orange; the Tn*6172* part or its partial segments are shown in blue, while the linker region is shown in green. Where possible, the backbone segments in the variants are placed in aligned positions. The names of the reported elements bearing the corresponding genetic configurations are given. NA, not available. Novel genetic configurations are shown in red. Dotted lines are used to connect sequences. Red blocks highlight the regions that might have gone through recombination, since they contained hybrid sequences from different backbones (for details, see Fig. S1 in the supplemental material). The schematic representations are drawn to scale.

Twenty-two complex configurations containing more than one backbone were all characterized as AbGRI1-type islands (Fig. 4 and 5). They displayed complex chimeric mosaicism. They had expanded variation profiles other than those of the solitary Tn*6022*- and Tn*6172-*derived elements (Table S2). In addition, IS*Aba17* and IS*Aba10* were found on the Tn*6022*-derived portions. Inversions of Tn*2006* were also observed. As for the Tn*6173*-derived part, there could be an insertion of a second Tn*6022*, Tn*6022*-derived, or even an AbGRI1-type element, splitting the 3′ end of the *tet*(B) gene that is located on the ΔIS*CR2*-ΔTn*10* segment, consistent with our previous observation that *tet*(B) was a high-frequency insertion site (4). We also found that the ΔIS*Pa14*-Tn*1213*-IS*Aba14*-*strA* region on the Tn*6173* part of AbaR4d (7) was variable, associated with the acquisition of *aph(3′)-VIb*. Moreover, a considerable fraction of AbGRI1-type configurations (Fig. 5) did not possess a typical Tn*6022*-linker-Tn*6172* structure, since the CSs from different backbones provide targets for homologous recombination, as illustrated by AbGRI1-5 (16). AbGRI1-5 featured a hybrid region made up of Tn*6022* and Tn*6173* segments. We further uncovered similar phenomena on AbaR26(BJAB0868) (17), AbaR25(BJAB07104) (17), and AbaR22 (18) (Fig. 5; see also Fig. S1 in the supplemental material). However, the precise boundaries of segments from different backbones were not specific and could hardly be defined. A hybrid region was not found on other atypical elements like AbaR4a/AbaGRI1-1, AbaR4b, and AbaR4c (7).

10.1128/mSphere.00349-20.1 FIG S1 Regions that might have gone through recombination contained hybrid sequences from different backbones. The regions that might have gone through recombination (denoted by red block) found in AbGRI1-5 (A), AbaR26(BJAB0868) (B), AbaR25(BJAB07104) (C), and AbaR22 (D) were aligned against Tn*6022* and Tn*6172*. Positions with consensus nucleotides are not shown. For each polymorphic position, nucleotides identical to those of Tn*6022* and Tn*6172* are shown with orange and blue shading, respectively. Download FIG S1, TIF file, 1.1 MB. Copyright © 2020 Bi et al. 2020 Bi et al. This content is distributed under the terms of the Creative Commons Attribution 4.0 International license.

10.1128/mSphere.00349-20.7 TABLE S2 Genetic configurations of the Tn*6022*- or Tn*6172*-derived variants that occurred only as part of an AbGRI1-type element. Download Table S2, DOCX file, 0.02 MB. Copyright © 2020 Bi et al. 2020 Bi et al. This content is distributed under the terms of the Creative Commons Attribution 4.0 International license.

### Structures of Tn*6661*, Tn*6662*, Tn*6663*, and Tn*6664*.

**FIG 6:**
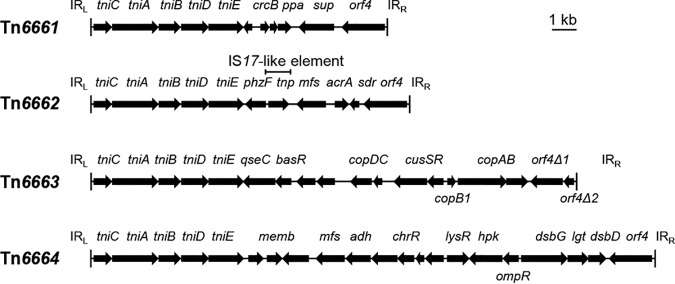
Schematic representations of the Tn*6661*, Tn*6662*, Tn*6663*, and Tn*6664* transposons that could not be mapped to the known backbones.

Tn*6661* (12.2 kb), Tn*6662* (13.1 kb), Tn*6663* (20.0 kb), and Tn*6664* (23.2 kb) were distinct elements (Fig. 6) showing 71% to 92% nucleotide identities to the known backbones (Fig. S2) and each other (Fig. S3) at the syntenic regions, covering the CSs at both ends. Like other backbone elements, they carried distinct genes in the middle regions. However, no antimicrobial resistance gene was identified. The Tn*6661* transposon harbors predicted *crcB* and *ppa* genes that might function as a fluoride transporter and an inorganic pyrophosphatase, respectively. Tn*6662* harbors genes that might participate in biosynthesis, as they were predicted to encode a PhzF family phenazine biosynthesis protein, a transporter, an oxidoreductase, and a transcriptional regulator. Tn*6663* carries a *cop* gene cluster likely involving in copper resistance. Tn*6664* carries a set of genes that might be linked to certain biochemical process, as they encoded putative enzymes such as disulfide reductase, dehydrogenase, oxidase, decarboxylase, and protein-disulfide reductase. Apart from an IS*17*-like element found on Tn*6662*, no other MGE was found on these elements, suggesting that they could serve as backbone structures. Interestingly, we found a Tn*6664*-type variant in Acinetobacter haemolyticus strains TJS01 (GenBank accession number CP018871; element coordinates, 3136595 to 3162983) (19). It had an IS*Aba8*-like IS and an IS*Ascp3*-like IS based on Tn*6664*. However, other variants based on these elements are yet to be revealed.

10.1128/mSphere.00349-20.2 FIG S2 Alignments of Tn*6661* (A), Tn*6662* (B), Tn*6663* (C), or Tn*6664* (D) with Tn*6019*, Tn*6022*, and Tn*6173*. Syntenic regions are connected by light orange. and the nucleotide-level identities are shown. The schematic representations are drawn to scale. Download FIG S2, TIF file, 0.9 MB. Copyright © 2020 Bi et al. 2020 Bi et al. This content is distributed under the terms of the Creative Commons Attribution 4.0 International license.

10.1128/mSphere.00349-20.3 FIG S3 Comparison among Tn*6661*, Tn*6662*, Tn*6663*, and Tn*6664*. Syntenic regions are connected by light orange, and the nucleotide-level identities are shown. The schematic representations are drawn to scale. Download FIG S3, TIF file, 0.5 MB. Copyright © 2020 Bi et al. 2020 Bi et al. This content is distributed under the terms of the Creative Commons Attribution 4.0 International license.

### The content and context characteristics of AbaRs were associated with backbones.

**FIG 7:**
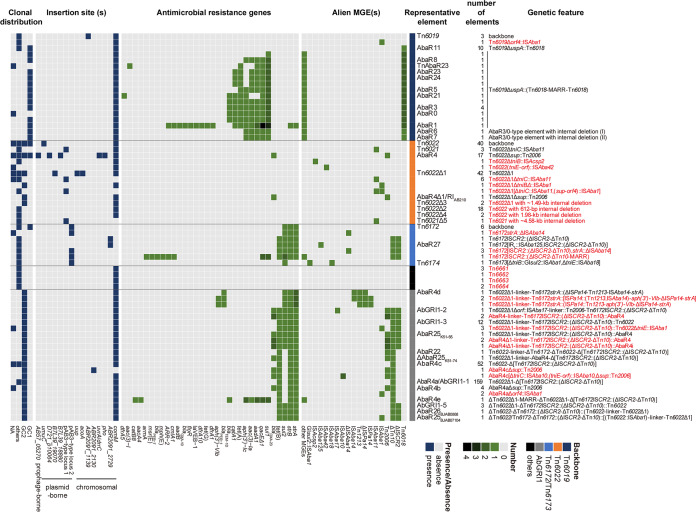
The acquired MGE pools, profiles of antimicrobial resistances genes, insertion sites, and clonal distribution are associated with backbones. The heatmaps are generated with the nonredundant elements. Novel genetic configurations are shown in red. See Data Set S1 for detailed information, including GenBank accession numbers, coordinates, and the mentioned features of all 442 analyzed elements. MGE, mobile genetic element; GC, global clone (see Fig. S4 for detailed sequence-type distribution). For the heatmap of alien MGEs, the IS*Aba1*, IS*CR2*, and ΔTn*5359* elements as parts of the Tn*6172* backbone and the IS*17*-like elements of Tn*6662* are not shown.

Finally, heatmaps profiling the acquired MGE pools, antimicrobial resistance gene profiles, insertion sites, and clonal distribution for the nonredundant AbaRs were generated (Fig. 7) and displayed apparent backbone-specific patterns. As mentioned above, the Tn*6019*-, Tn*6022*-, Tn*6172*/Tn*6173*-, and AbGRI1-type variants were associated with different sets of acquired MGEs. Moreover, they carried distinct spectrums of antimicrobial resistance genes that were specific to the backbones. They also showed differences in insertion sites and clonal distributions. Consistent with previous reports (3, 16), the Tn*6019*- and AbGRI1-type elements were mostly associated with the conventional *comM* insertion site, with very few exceptions in other loci, and almost confined to specific clonal lineages, GC1 and GC2, respectively. However, the Tn*6022*-type AbaRs had more diverse insertion sites and wider clonal distribution (Fig. 7 and Fig. S4) than the other types. The Tn*6172*/Tn*6173*-type AbaRs were associated with a plasmid-borne insertion site instead of the *comM*. The Tn*6661*, Tn*6662*, Tn*6663*, and Tn*6664* tranposons were found in minor clones, and their features need further investigation, as there might be sample bias in the current genome database.

10.1128/mSphere.00349-20.4 FIG S4 Distribution of the genetic configuration of in clones with different sequence types. Sequence types (STs) were based on the Pasteur multilocus sequence typing scheme (L. Diancourt, V. Passet, A. Nemec, L. Dijkshoorn, and S. Brisse, PLoS One 5:e10034, 2010, https://doi.org/10.1371/journal.pone.0010034), and clone lineage information is shown in parentheses if available, which was based on the trilocus typing scheme (J. F. Turton, S. N. Gabriel, C. Valderrey, M. E. Kaufmann, and T. L. Pitt, Clin Microbiol Infect 13:807−815, 2007, https://doi.org/10.1111/j.1469-0691.2007.01759.x) or eBURST (E. J. Feil, B. C. Li, D. M. Aanensen, W. P. Hanage, and B. G. Spratt, J Bacteriol 186:1518−1530, 2004, https://doi.org/10.1128/jb.186.5.1518-1530.2004) analysis. Novel genetic configurations are shown in red. Download FIG S4, TIF file, 1.1 MB. Copyright © 2020 Bi et al. 2020 Bi et al. This content is distributed under the terms of the Creative Commons Attribution 4.0 International license.

## DISCUSSION

AbaRs are prevalent in A. baumannii. We had previously performed large-scale identification of AbaRs in A. baumannii genomes and found that their antimicrobial resistance gene profiles were specific to clonal lineage (4). However, the exact genetic configurations involving backbone types and variation patterns of the identified elements remained unknown. Here, we conducted further comparative genomic analysis of AbaRs. We uncovered novel genetic configurations of AbaRs and found that the content and context characteristics of AbaRs were specific to backbones.

The newly identified genetic configurations mainly involved insertions of novel MGEs or novel structural modulations driven by known MGEs. We found a rare Tn*6019*-derived configuration that carried an IS*Aba1*. IS*Aba1* is an active IS in A. baumannii and has been widely found in this species. As for AbaRs, the IS*Aba1* has been seen on AbaR4, Tn*6172*, and some AbGRI1-type elements as part of Tn*2006* or alone (5, 7, 20) but has not yet been reported as occurring on Tn*6019*. Tn*2006* is closely associated with the variations based on Tn*6022*, as it has been frequently seen on Tn*6022*-type elements or the Tn*6022*-derived parts of AbGRI1-type elements (7, 13, 21, 22). Meanwhile, diverse backbone deletions were found on Tn*6022*, but the mechanisms of the variations remain unknown. IS*CR2* might play a role in modulating the plasticity of Tn*6172* and Tn*6173*, since the ΔIS*CR2*-ΔTn*10* fragment or a larger fragment containing that structure was often found at the IS*CR2* locus (8). Moreover, in AbGRI1-type elements, the *tet*(B) gene on the ΔIS*CR2*-ΔTn*10* fragment provided a hot spot for insertion of another AbaR, resulting in complex chimeric structures. This type of configuration was found on reported elements like RI_MDT-TJ_/AbGRI1-3 (14) and AbaR26(BJAB0868) (17), but it had not been previously elucidated. It seems the multiple homologous backbone CSs in the AbGRI1-type elements have actively triggered complex recombination events, generating diverse atypical AbGRI1-type elements containing hybrid backbone sequences. Although the genetic events behind this process remain unclear, it is undoubted that this nature could dramatically complicate the evolution of AbGRI1-type elements.

A comprehensive profiling of the genetic features of AbaRs uncovered backbone-specific patterns. The acquired MGE pools of AbaRs remarkably differed by backbone types, which directly resulted in distinct antimicrobial resistance gene profiles, since the acquired resistance determinants were almost associated with the acquired MGEs. For example, the multiple antimicrobial resistance of Tn*6019*-type AbaRs was attributed to the Tn*6018* compound elements (3), the only resistance gene that appeared on Tn*6022*-type AbaRs, *bla*_OXA-23_, was embedded in Tn*2006* (23), and the *tet*(B), *bla*_PER-1_, or other resistance genes on Tn*6172*/Tn*6173* were cargo of ΔTn*10*, Tn*1213*, or IS*CR2*-associated regions, respectively (7, 8). Notably, on the CS regions, AbaRs still had backbone-specific structural variations. The variation might be due to the fact that the CSs of different backbones had nucleotide variations, some of which might result in favorable targeting sequences for certain MGEs, or to the possibility that the AbaRs might have experienced different MGE exposure in different clones. Indeed, AbaRs of most backbone types are associated with specific clonal lineages. We have previously observed the association between antimicrobial resistance gene profiles and clonal distribution (4); here, we were able to extend the correlation with backbone types. We have also reported that AbaRs have diverse insertion sites other than *comM* (4). Here, we further demonstrated that this feature was mainly found in Tn*6022*-type AbaRs. In addition, it is likely that plasmids might promote the interclonal exchange of AbaRs (24), since the Tn*6022*-type and Tn*6172*/Tn*6173*-type AbaRs spanning multiple clonal lineages (Fig. 7; see also Fig. S4 in the supplemental material) both had plasmid-borne insertion sites. However, the AbGRI1-type islands did not share this feature and were mostly found in GC2. These results indicated that AbaRs with different backbones might have evolved separately.

The term “AbaR” initially referred to resistance islands in A. baumannii (1) but has also been used for the closely related *comM*-associated elements described above, regardless of whether they carry a resistance gene. It also should be noted that these elements are not confined to just the *comM* locus. The term “AbaR” has certain limitations, since these elements display wide diversity. It would be better if the nomenclature were based on the transposition module and/or the transposition process of these elements. Given the backbone-specific nature of these elements revealed by this study, we propose that the elements with different backbones be treated separately, and that using backbone-based nomenclature or specified Tn (transposon) numbers could be more specific and informative than the generic term “AbaR” to reflect the genetic features of these elements. We envision that this study could help lead to a solution on the nomenclature of these elements.

Overall, this study uncovered genetic features of “AbaRs,” including the profiles of the MGEs driving the plasticity of these elements and the consequently acquired antimicrobial resistance genes, clonal distribution, as well as insertion sites displaying strong associations with their backbones.

## MATERIALS AND METHODS

### Genomic data of AbaRs.

Information on 468 intact AbaRs from our recent identification of A. baumannii genomes (4), literature, and unpublished entries in the GenBank database were collected, and 442 were included for analysis (see Data Set S1 in the supplemental material). Among the excluded AbaRs, 21 contained large sequencing gaps, and five turned out to be portions of AbGRI1-type elements with incomplete sequences rather than individual elements. Specifically, the last five were initially identified on contigs as intact elements (Tn*6022*) (4), but further analysis here showed that their upstream and downstream sequences could be mapped to the Tn*6172* backbone, which meant that the identified elements were actually parts of AbGRI1-type elements. There was not enough sequence information on the corresponding contigs to recognize the entire elements with the previously described algorithm (4). Among the included AbaRs, 36 have been previously characterized, 402 were recently predicted, and 4 were from unpublished GenBank entries (Data Set S1). The latter two kinds have not been characterized before.

### Comparative analysis and genetic configuration profiling of AbaRs.

AbaRs were mapped to backbones Tn*6019* (5), Tn*6022* (9), Tn*6172* (10), Tn*6173* (8), and AbGRI1-0 (8) via BLAST with a cutoff of >99% overall nucleotide identities (or >90% for elements with the Tn*6022*Δ2 configuration). Alien sequences were characterized as previously described (11). Genetic configurations were defined manually. Complex elements (*n* = 253) containing multiple backbones were also aligned with the elements (*n* = 189) containing one backbone for comparison. A nonredundant set of the AbaRs was generated with a cutoff of >99% coverage and >98% identity (or >95% for the Tn*6022*Δ2 configuration) for analysis.

### Multiple-sequence alignment.

Multiple-sequence alignment was performed with MUSCLE (25) to identify hybrid regions from different backbones.

### Heatmaps.

Heatmaps were generated with the ComplexHeatmap package (26) in R.

### Accession number(s).

The accession numbers of all sequences used in this study are listed in Data Set S1 in the supplemental material along with precise coordinates where applicable.
