# Supplementary material for: Comparative Analysis of AbaR-Type Genomic Islands Reveals Distinct Patterns of Genetic Features in Elements with Different Backbones
Source: mSphere. 2020 May 27;5(3):e00349-20. doi: 10.1128/mSphere.00349-20 (PMC7253598; doi:10.1128/mSphere.00349-20)
Supplement: TABLE S1 [file mSphere.00349-20-st001.docx]

Table S1. Genetic configurations of AbaR-type genomic islands and their features.

| Backbone | Genetic configuration^a^ | Size (bp)^b^ | GC^b^ | Reported elements | MGE(s) on the backbone | Antimicrobial resistance genes | Insertion site^c^ | ST(clone lineage)^d^ | Ref |
| --- | --- | --- | --- | --- | --- | --- | --- | --- | --- |
| Tn*6019* | backbone | 16332 | 37.8% | Tn*6019* |  |  | *comM*, *ABR2091_1139* | ST128, ST32 | ([1](#_ENREF_1)) |
|  | Tn*6019*Δ*orf4*::IS*Aba1* | 17521 | 37.7% |  | IS*Aba1* |  | *comM* | ST32 | This study |
|  | Tn*6019*Δ*uspA*::Tn*6018* | 19713(±1) | 40.8% | AbaR11 | Tn*6018* |  | *comM* | ST1(GC1), ST255 | ([2](#_ENREF_2), [3](#_ENREF_3)) |
|  | Tn*6019*Δ*uspA*::(Tn*6018*-MARR-Tn*6018*)  (refer to a class of variants with variable MARRs) | 31127-86244 | 48.2%~52.8% | AbaR3/0-type elements | Tn*6018*, variable and diverse MGEs within the two copies of Tn*6018* | gene pool^e^: *strA*, *strB*, *dfrA1*, *qacEΔ1*, *floR*, *tet*(G), *bla*_VEB-1_, *aadB*, *ARR-2*, *cmlA1*, *bla*_OXA-10_, *aadA1*, *sul1*, *dfrA10*, *tet*(A), *catA1*, *aph(3')-Ic*, *aac(3)-Ia*, *bla*_TEM-1D_, *dfrA5*, *aac(3)-I* | *comM* | ST1(GC1) | ([2](#_ENREF_2), [3](#_ENREF_3)) |
|  | AbaR3/0-type element with internal deletion^f^ | 27392 | 47.7% | AbaR6 | Tn*6018*, MGEs associated with the MARR | *aph(3')-Ic*, *aac(3)-Ia*, *aadA1*, *qacEΔ1*, *sul1* | *comM* | (GC1) | ([1](#_ENREF_1)) |
|  | AbaR3/0-type element with internal deletion^f^ | 19668 | 50.9% | AbaR7 | Tn*6018*, MGEs associated with the MARR | *aph(3')-Ic*, *aac(3)-Ia*, *aadA1*, *qacEΔ1*, *sul1* | *comM* | (GC1) | ([1](#_ENREF_1)) |
| Tn*6022* | backbone | 11998  (-1/-2) | 36.1%  (-0.1%) | Tn*6022* |  |  | *comM*, *umuC*, *tet*(B) | ST2(GC2), ST15(Clonal Group 5), ST19(GC1), ST32, ST40, ST46, ST79, ST85, ST422, ST499 | ([4](#_ENREF_4)) |
|  | Tn*6022*Δ*tniC*::IS*Aba11* | 13103(+1) | 35.9% | Tn*6021* | IS*Aba11* |  | *comM*, pAB3-type locus 1 | ST437 | ([1](#_ENREF_1)) |
|  | Tn*6022*Δ*sup*::Tn*2006* | 16812  (-6~+2) | 36.3%  (-0.1%) | AbaR4 | Tn*2006* | *bla*_OXA-23_ | *comM*, *pho*, *B7L39_18880*, *uvrA*/*drrC*, *AB57_05270*, *D721_p10064*, *tet*(B) | ST1(GC1), ST2(GC2), ST10, ST25 (Clonal Group 4), ST81 (GC1), ST149, ST622 | ([5](#_ENREF_5)) |
|  | Tn*6022*Δ*tniB*::IS*Acsp2* | 12979 | 36.0% |  | IS*Acsp2* |  | *comM* | ST422 | This study |
|  | Tn*6022*(*tniE*-*orf*)::IS*Aba42* | 13326 | 36.7% |  | IS*Aba42* |  | *comM* | NA | This study |
|  | Tn*6022*Δ1 | 9148  (-2/-5) | 35.7%  (+0.1%) | Tn*6022*Δ1 |  |  | *comM*, *uup*, *acoA*, *B7L39_19070* | ST2(GC2), ST187 (GC2), ST215 | ([6](#_ENREF_6)) |
|  | Tn*6022*Δ1Δ*tniC*::IS*Aba11* | 10254(-2) | 35.6% |  | IS*Aba11* |  | pAB3-type locus 1 | ST10, ST25 (Clonal Group 4), ST494 | This study |
|  | Tn*6022*Δ1Δ*tniBΔ*::IS*Aba1* | 10337 | 35.7% |  | IS*Aba1* |  | *comM* | ST2(GC2) | This study |
|  | Tn*6022*Δ1[Δ*tniC*::IS*Aba11*,(*sup*-*orf4*)::IS*Aba1*] | 11443 | 35.6% |  | IS*Aba11*, IS*Aba1* |  | pAB3-type locus 1 | ST25 (Clonal Group 4) | This study |
|  | Tn*6022*Δ1Δ*sup*::Tn*2006* | 13962 | 36.1% | RI_AB210_/AbaR4Δ1^g^ | Tn*2006* | *bla*_OXA-23_ | *comM* | (GC2) | ([7](#_ENREF_7)) |
|  | Tn*6022*Δ1 with ~1.49-kb internal deletion | 7661 | 34.9% |  |  |  | *comM* | ST2(GC2) | This study |
|  | Tn*6022* with 612-bp internal deletion | 11388(+1) | 36.5% |  |  |  | *comM* | ST39, ST229, ST431, ST500 (Clonal Group 3), ST521, ST537, ST973 | This study |
|  | Tn*6022* with ~1.98-kb internal deletion | 10021 | 35.9% |  |  |  | *comM* | ST40 | This study |
|  | Tn*6021* with ~4.58-kb internal deletion | 8529 | 35.9% |  | IS*Aba11* |  | pAB3-type locus 1 | ST126 | This study |
| Tn*6172* | backbone | 11719(-1) | 45.0% | Tn*6172* |  | *sul2*, *strB*, *strA* | pAB3-type locus 2 | ST1(GC1), ST3(Clonal Group 3), ST25(Clonal Group 4) | ([8](#_ENREF_8)) |
|  | Tn*6172strA*::ΔIS*Aba14* | 11994 | 44.9% |  | ΔIS*Aba14* | *sul2*, *strB*, *strA* | pAB3-type locus 2 | NA | This study |
|  | Tn*6172*IS*CR2*::(ΔIS*CR2*-ΔTn*10*) | 14299 | 44.6% |  | ΔIS*CR2*, ΔTn*10* | *sul2*, *tet*(B), *strB*, *strA* | *ABR2091_2729* | ST23 | ([9](#_ENREF_9)) |
|  | Tn*6172*[IR_L_::IS*Aba125*,IS*CR2*::(ΔIS*CR2*-ΔTn*10*)] | 15389 | 44.0% | AbaR27 | IS*Aba125*, ΔIS*CR2*, ΔTn*10* | *sul2*, *tet*(B), *strB*, *strA* | *ABR2091_2729* | ST23 | ([10](#_ENREF_10)) |
|  | Tn*6172*[ISCR2::(ΔISCR2-ΔTn10),*strA*::ΔIS*Aba14*] | 14574  (-39) | 44.5%  (-0.1%) |  | ΔIS*CR2*, ΔTn*10*, ΔIS*Aba14* | *sul2*, *tet*(B), *strB*, *strA* | pAB3-type locus 2 | ST25(Clonal Group 4) | This study |
|  | Tn*6172*IS*CR2*::(ΔIS*CR2*-ΔTn*10*-MARR) | 57246 | 48.7% |  | ΔIS*CR2*, ΔTn*10*, diverse MGEs | *sul2*, *tet*(B), *mph*(E), *msr*(E), *armA*, *sul1*, *bla*_PER-7_, *qacEΔ1*, *cmlA1*, *ARR-2*, *strB*, *strA* | pAB3-type locus 2 | ST10 | This study |
| Tn*6173* | backbone (hypothetical transposon) | 12370^h^ | 36.9%^h^ |  |  |  |  |  | ([9](#_ENREF_9)) |
|  | Tn*6173*[Δ*tniB*::GI*sul2*::IS*Aba1*,Δ*tniE*::IS*Aba18*] | 30331 | 47.5% | Tn*6174* | GI*sul2*::IS*Aba1*, IS*Aba18* | *sul2* | pAB3-type locus 2 | ST437 | ([9](#_ENREF_9)) |
| Tn*6661* |  | 12181 | 36.2% |  |  |  | *comM* | ST39, ST240, ST138 | This study |
| Tn*6662* |  | 13095 | 35.8% |  |  |  | *comM* | ST923 | This study |
| Tn*6663* |  | 19969 | 35.8% |  |  |  | *comM* | ST1166 | This study |
| Tn*6664* |  | 23184 | 38.0% |  |  |  | *comM* | ST529 | This study |
| AbGRI1-0 | Tn*6022*-linker-Tn*6172*  (ancestral form of AbGRI1 islands; hypothetical transposon) | 29922^i^ | 38.9%^i^ | AbGRI1-0 |  | *sul2*, *strB*, *strA* |  |  | ([9](#_ENREF_9)) |
|  | Tn*6022*Δ1-linker-Tn*6172strA*::(ΔIS*Pa14*-Tn*1213*-IS*Aba14*-*strA*) | 33988 | 40.2% | AbaR4d | ΔIS*Pa14*, Tn*1213*, IS*Aba14* | *sul2*, *strB*, *strA*, *bla*_PER-1_ | *comM* | ST2 (GC2) | ([11](#_ENREF_11)) |
|  | Tn*6022*Δ1-linker-Tn*6172strA*::[IS*Pa14*::(Tn*1213*,IS*Aba14*)-*aph(3')-VIb*-ΔIS*Pa14*-*strA*] | 36001 | 39.7% |  | ΔIS*Pa14*, IS*Pa14*, Tn*1213*, IS*Aba14* | *sul2*, *strB*, *strA*, *bla*_PER-1_, *aph(3')-VIb* | *comM* | ST2 (GC2) | This study |
|  | Tn6022Δ1-linker-Tn*6172strA*::(IS*Pa14*::Tn1213-*aph(3')-VIb*-ΔIS*Pa14*-*strA*) | 34717 | 39.8% |  | ΔIS*Pa14*, IS*Pa14*, Tn*1213* | *sul2*, *strB*, *strA*, *bla*_PER-1_, *aph(3')-VIb* | *comM* | ST2 (GC2) | This study |
|  | Tn*6022*Δ1Δ*orf*::IS*Aba17*-linker::Tn*2006*-Tn*6172*IS*CR2*::(ΔIS*CR2*-ΔTn*10*) | 37068 | 39.6% | AbGRI1-2/ Tn*6167* | IS*Aba17*, Tn*2006*, ΔIS*CR2*, ΔTn*10* | *bla*_OXA-23_, *sul2*, *tet*(B), *strB*, *strA* | *comM* | (GC2) | ([12](#_ENREF_12)) |
|  | AbaR4-linker-Tn*6172*IS*CR2*::(ΔIS*CR2*-ΔTn*10*)::AbaR4 | 54133 | 38.1% |  | Tn*2006*, ΔIS*CR2*, ΔTn*10* | *bla*_OXA-23_, *sul2*, *tet*(B), *strB*, *strA* | *comM* | ST2 (GC2) | This study |
|  | Tn*6022*Δ1-linker-Tn*6172*IS*CR2*::(ΔIS*CR2*-ΔTn*10*)::Tn*6022* | 41655  (-12~+3) | 38.5% | AbGRI1-3/RI_MDR-TJ_, RI_TYTH-1_ | ΔIS*CR2*, ΔTn*10* | *sul2*, *tet*(B), *strB*, *strA* | *comM* | ST2 (GC2) | ([13](#_ENREF_13), [14](#_ENREF_14)) |
|  | Tn*6022*Δ1-linker-Tn*6172*IS*CR2*::(ΔIS*CR2*-ΔTn*10*)::Tn*6022*Δ*tniE*::IS*Aba1* | 42844 | 38.4% |  | ΔIS*CR2*, ΔTn*10*, IS*Aba1* | *sul2*, *tet*(B), *strB*, *strA* | *comM* | ST2 (GC2) | This study |
|  | Tn*6022*Δ1-linker-Tn*6172*IS*CR2*::(ΔIS*CR2*-ΔTn*10*)::AbaR4 | 46469 | 38.3% | AbaR25(K51-65)^j^ | ΔIS*CR2*, ΔTn*10*, Tn*2006* | *sul2*, *bla*_OXA-23_, *tet*(B), *strB*, *strA* | *comM* | ST2 (GC2) | ([15](#_ENREF_15)) |
|  | AbaR4Δ1-linker-Tn*6172*IS*CR2*::(ΔIS*CR2*-ΔTn*10*)::AbaR4 | 51283 | 38.2%  (-0.1%) |  | ΔIS*CR2*, ΔTn*10*, Tn*2006* | *bla*_OXA-23_, *sul2*, *tet*(B), *strB*, *strA* | *comM* | ST2 (GC2) | This study |
|  | AbaR4iΔ1-linker-Tn*6172*IS*CR2*::(ΔIS*CR2*-ΔTn*10*)::AbaR4i^l^ | 51283 | 38.2% |  | ΔIS*CR2*, ΔTn*10*, Tn*2006* | *bla*_OXA-23_, *sul2*, *tet*(B), *strB*, *strA* | *comM* | ST2 (GC2) | This study |
|  | Tn*6022*-linker-ΔTn*6172*-ΔTn*6022*-Δ[Tn*6172*IS*CR2*::(ΔIS*CR2*-ΔTn*10*)] | 38683 | 37.8% | AbaR22 | ΔTn*10* | *tet*(B), *strB*, *strA* | *comM* | ST2 (GC2) | ([16](#_ENREF_16)) |
|  | Tn*6022*Δ1-linker-AbaR4-Δ[Tn*6172*IS*CR2*::(ΔIS*CR2*-ΔTn*10*)] | 40647 | 37.8% | ΔAbaR25(K51-74) | ΔTn*10*, Tn*2006* | *bla*_OXA-23_, *tet*(B), *strB*, *strA* | *comM* | (GC2) | ([15](#_ENREF_15)) |
|  | Tn*6022*-Δ[Tn*6172*IS*CR2*::(ΔIS*CR2*-ΔTn*10*)] | 20480(-34~+10) | 40.3% | AbaR4c | ΔTn*10* | *tet*(B), *strB*, *strA* | *comM* | ST2(GC2), ST215, ST499 | ([11](#_ENREF_11)) |
|  | AbaR4cΔ*sup*::Tn*2006* | 25279 | 39.7% |  | Tn*2006*, ΔTn*10* | *bla*_OXA-23_*, *tet*(B), *strB*, *strA* | *ABR2091_2130* | ST2 (GC2) | This study |
|  | AbaR4c[Δ*tniC*::IS*Aba10*,(*tniE*-*orf*)::IS*Aba10*,Δ*sup*::Tn*2006*] | 27355 | 39.8% |  | IS*Aba10*, Tn*2006*, ΔTn*10* | *bla*_OXA-23_, *tet*(B), *strB*, *strA* | *comM* | ST2 (GC2) | This study |
|  | Tn*6022*Δ1-Δ[Tn*6172*IS*CR2*::(ΔIS*CR2*-ΔTn*10*)] | 17630 | 40.9%  (-0.1%) | AbaR4a, AbGRI1-1/Tn*6166* | ΔTn*10* | *tet*(B), *strB*, *strA* | *comM* | ST2(GC2), ST215, ST922(GC2), ST1088 | ([6](#_ENREF_6), [11](#_ENREF_11)) |
|  | AbaR4aΔ*sup*::Tn*2006* | 22444(+2) | 40.0% | AbaR4b | Tn*2006*, ΔTn*10* | *bla*_OXA-23_, *tet*(B), *strB*, *strA* | *comM* | ST2 (GC2) | ([11](#_ENREF_11)) |
|  | AbaR4aΔ*orf4*::IS*Aba1* | 18822 | 40.5% |  | IS*Aba1*, ΔTn*10* | *tet*(B), *strB*, *strA* | *comM* | ST2 (GC2) | This study |
|  | ΔTn*6022*Δ1-MARR-ΔTn*6022*Δ1-Δ[Tn*6172*IS*CR2*::(ΔIS*CR2*-ΔTn*10*)] | 49581 | 48.5% | AbaR4e | ΔTn*10*, diverse MGEs within the MARR | *bla*_TEM-1D_, *aph(3')-Ic*, *aacA4*, *catB8*, *aadA1*, *qacEΔ1*, *sul1*, *aph(3')-Ic*, *aac(3)-Ia*, *tet*(B), *strB*, *strA* | *comM* | ST2 (GC2) | ([11](#_ENREF_11)) |
|  | ΔTn*6022*Δ1-ΔTn*6172*IS*CR2*::(ΔIS*CR2*-ΔTn*10*)::Tn*6022* | 26302(-1) | 40.8% | AbGRI1-5 | ΔIS*CR2*, ΔTn*10* | *sul2*, *tet*(B), *strB*, *strA* | *comM* | ST2 (GC2) | ([17](#_ENREF_17)) |
|  | ΔTn6022-ΔTn*6172*::ΔIS*CR2*-ΔTn*10*)::(Tn*6022*-linker-Tn*6022*Δ1) | 41656 | 38.5% | AbaR26(BJAB0868)^k^ | ΔIS*CR2*, ΔTn*10* | *sul2*, *tet*(B), *strB*, *strA* | *comM* | ST2 (GC2) | ([10](#_ENREF_10)) |
|  | ΔTn6022/Tn6172-Δ(Tn*6172*::ΔIS*CR2*-ΔTn*10*)::[(Tn*6022*::IS*Aba1*)-linker-Tn*6022*Δ1] | 42847 | 38.4% | AbaR25(BJAB07104)^j^ | ΔIS*CR2*, ΔTn*10*, IS*Aba1* | *sul2*, *tet*(B), *strB*, *strA* | *comM* | ST2 (GC2) | ([10](#_ENREF_10)) |

GC, GC content; MGE, mobile genetic element; ST, sequence type; Ref, reference; MARR, multiple antimicrobial resistance region. GC, global clone *, frame-shifted

^a^ See Figs 1-6 for detailed schematic illustration.

^b^ Elements with a same configuration may have slightly varied length and GC contents, in this case, the length and GC content of the majority of the elements are given and the deviations for the minority are shown in the brackets.

^c^ For a name-unassigned gene, a representative locus tag identifier of an identical undisrupted gene is given, which is consistent with our previous report (Bi et al. Antimicrobial Agents and Chemotherapy, 2019, 63:e02526-18).

^d^ Sequence types are based on the Pasteur multi-locus sequence typing scheme ([18](#_ENREF_18)) and clone lineage information is shown in the brackets if available, which is based on the tri-locus typing scheme ([19](#_ENREF_19)) or eBURST ([20](#_ENREF_20)) analysis.

^e^ A non-redundant list of the antimicrobial resistance genes of a class of variants with the Tn*6019*Δ*uspA*::(Tn*6018*-MARR-Tn*6018*) configuration.

^f^ With different extents of deletion on the backbones.

^g^ Designated AbaR4Δ1 by this study.

^h^ Data extracted from Tn*6174*.

^I^ Estimated data.

^j^ AbaR25(BJAB07104) and AbaR25(K51-65) are different elements.

^k^ AbaR26(BJAB0868) is different to a reported truncated element also termed AbaR26 found in strain D30 (KC665626).

^l^ The letter "i" in the names "AbaR4iΔ1" and "AbaR4i" means the Tn*2006* in AbaR4iΔ1 and AbaR4i are inverted when comparing to that in AbaR4Δ1 and AbaR4, respectively.

**References for Table S1**

1. Post V, White PA, Hall RM. 2010. Evolution of AbaR-type genomic resistance islands in multiply antibiotic-resistant *Acinetobacter baumannii*. J Antimicrob Chemother 65:1162-70.

2. Hamidian M, Hall RM. 2018. The AbaR antibiotic resistance islands found in *Acinetobacter baumannii* global clone 1 - Structure, origin and evolution. Drug Resist Updat 41:26-39.

3. Krizova L, Dijkshoorn L, Nemec A. 2011. Diversity and evolution of AbaR genomic resistance islands in *Acinetobacter baumannii* strains of European clone I. Antimicrob Agents Chemother 55:3201-6.

4. Hamidian M, Hall RM. 2011. AbaR4 replaces AbaR3 in a carbapenem-resistant *Acinetobacter baumannii* isolate belonging to global clone 1 from an Australian hospital. J Antimicrob Chemother 66:2484-91.

5. Adams MD, Goglin K, Molyneaux N, Hujer KM, Lavender H, Jamison JJ, MacDonald IJ, Martin KM, Russo T, Campagnari AA, Hujer AM, Bonomo RA, Gill SR. 2008. Comparative genome sequence analysis of multidrug-resistant *Acinetobacter baumannii*. J Bacteriol 190:8053-64.

6. Nigro SJ, Hall RM. 2012. Antibiotic resistance islands in A320 (RUH134), the reference strain for *Acinetobacter baumannii* global clone 2. J Antimicrob Chemother 67:335-8.

7. Turton JF, Baddal B, Perry C. 2011. Use of the accessory genome for characterization and typing of *Acinetobacter baumannii*. J Clin Microbiol 49:1260-6.

8. Hamidian M, Hall RM. 2016. The resistance gene complement of D4, a multiply antibiotic-resistant ST25 *Acinetobacter baumannii* isolate, resides in two genomic islands and a plasmid. J Antimicrob Chemother 71:1730-2.

9. Hamidian M, Hall RM. 2017. Origin of the AbGRI1 antibiotic resistance island found in the *comM* gene of *Acinetobacter baumannii* GC2 isolates. J Antimicrob Chemother 72:2944-2947.

10. Zhu L, Yan Z, Zhang Z, Zhou Q, Zhou J, Wakeland EK, Fang X, Xuan Z, Shen D, Li QZ. 2013. Complete genome analysis of three *Acinetobacter baumannii* clinical isolates in China for insight into the diversification of drug resistance elements. PLoS One 8:e66584.

11. Seputiene V, Povilonis J, Suziedeliene E. 2012. Novel variants of AbaR resistance islands with a common backbone in *Acinetobacter baumannii* isolates of European clone II. Antimicrob Agents Chemother 56:1969-73.

12. Nigro SJ, Hall RM. 2012. Tn*6167*, an antibiotic resistance island in an Australian carbapenem-resistant *Acinetobacter baumannii* GC2, ST92 isolate. J Antimicrob Chemother 67:1342-6.

13. Huang H, Yang ZL, Wu XM, Wang Y, Liu YJ, Luo H, Lv X, Gan YR, Song SD, Gao F. 2012. Complete genome sequence of *Acinetobacter baumannii* MDR-TJ and insights into its mechanism of antibiotic resistance. J Antimicrob Chemother 67:2825-32.

14. Liu CC, Tang CY, Kuo HY, Lu CW, Chang KC, Liou ML. 2013. The origin of *Acinetobacter baumannii* TYTH-1: a comparative genomics study. Int J Antimicrob Agents 41:318-24.

15. Saule M, Samuelsen O, Dumpis U, Sundsfjord A, Karlsone A, Balode A, Miklasevics E, Karah N. 2013. Dissemination of a carbapenem-resistant *Acinetobacter baumannii* strain belonging to international clone II/sequence type 2 and harboring a novel AbaR4-like resistance island in Latvia. Antimicrob Agents Chemother 57:1069-72.

16. Zhou H, Zhang T, Yu D, Pi B, Yang Q, Zhou J, Hu S, Yu Y. 2011. Genomic analysis of the multidrug-resistant *Acinetobacter baumannii* strain MDR-ZJ06 widely spread in China. Antimicrob Agents Chemother 55:4506-12.

17. Nigro SJ, Brown MH, Hall RM. 2019. AbGRI1-5, a novel AbGRI1 variant in an *Acinetobacter baumannii* GC2 isolate from Adelaide, Australia. J Antimicrob Chemother 74:821-823.

18. Diancourt L, Passet V, Nemec A, Dijkshoorn L, Brisse S. 2010. The population structure of *Acinetobacter baumannii*: expanding multiresistant clones from an ancestral susceptible genetic pool. PLoS One 5:e10034.

19. Turton JF, Gabriel SN, Valderrey C, Kaufmann ME, Pitt TL. 2007. Use of sequence-based typing and multiplex PCR to identify clonal lineages of outbreak strains of *Acinetobacter baumannii*. Clin Microbiol Infect 13:807-15.

20. Feil EJ, Li BC, Aanensen DM, Hanage WP, Spratt BG. 2004. eBURST: inferring patterns of evolutionary descent among clusters of related bacterial genotypes from multilocus sequence typing data. J Bacteriol 186:1518-30.
