# Supplementary figures and images for: Comparative Analysis of AbaR-Type Genomic Islands Reveals Distinct Patterns of Genetic Features in Elements with Different Backbones
Source: mSphere. 2020 May 27;5(3):e00349-20. doi: 10.1128/mSphere.00349-20 (PMC7253598; doi:10.1128/mSphere.00349-20)

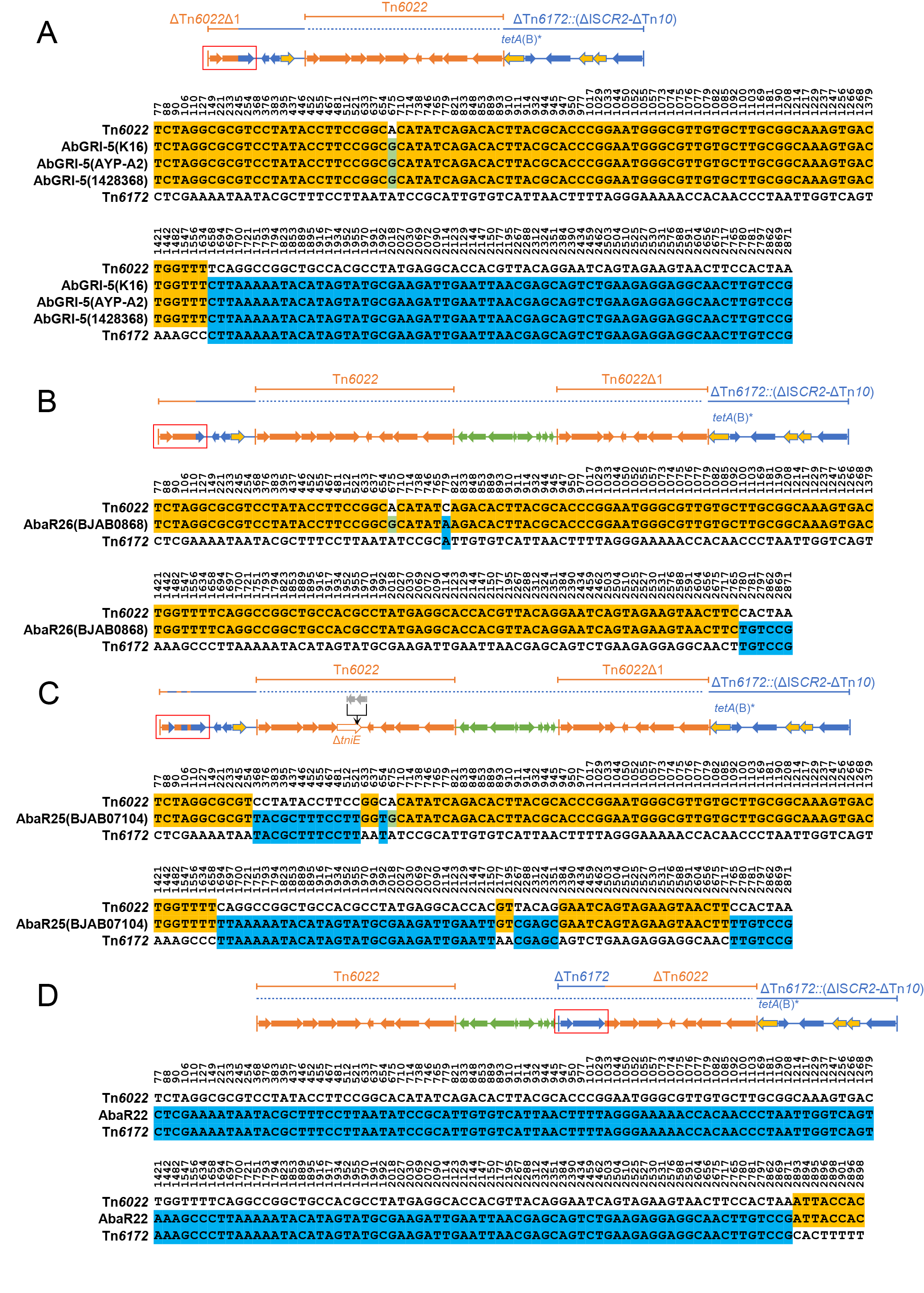

Supplement: FIG S1 [file mSphere.00349-20-sf001.tif]

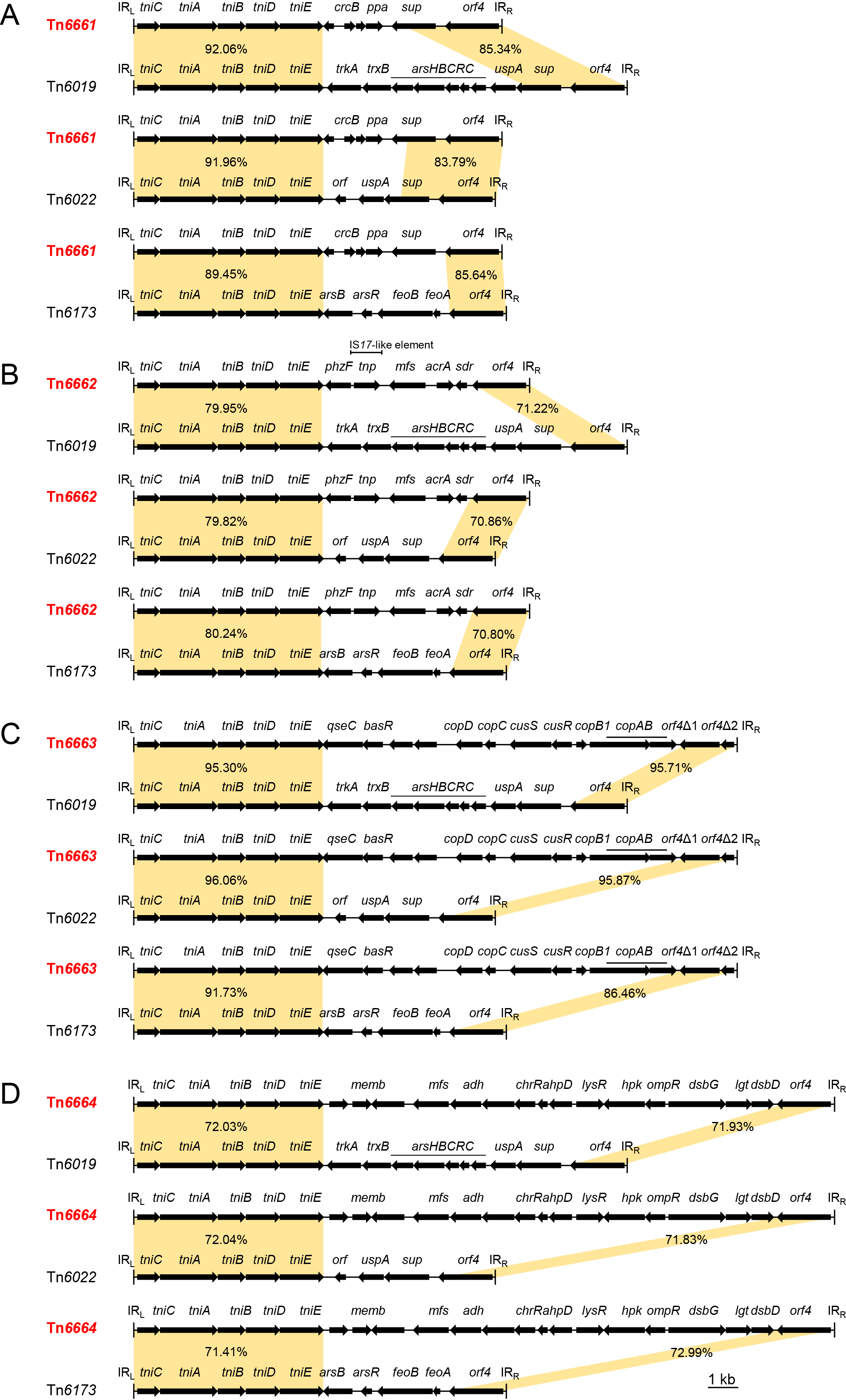

Supplement: FIG S2 [file mSphere.00349-20-sf002.tif]

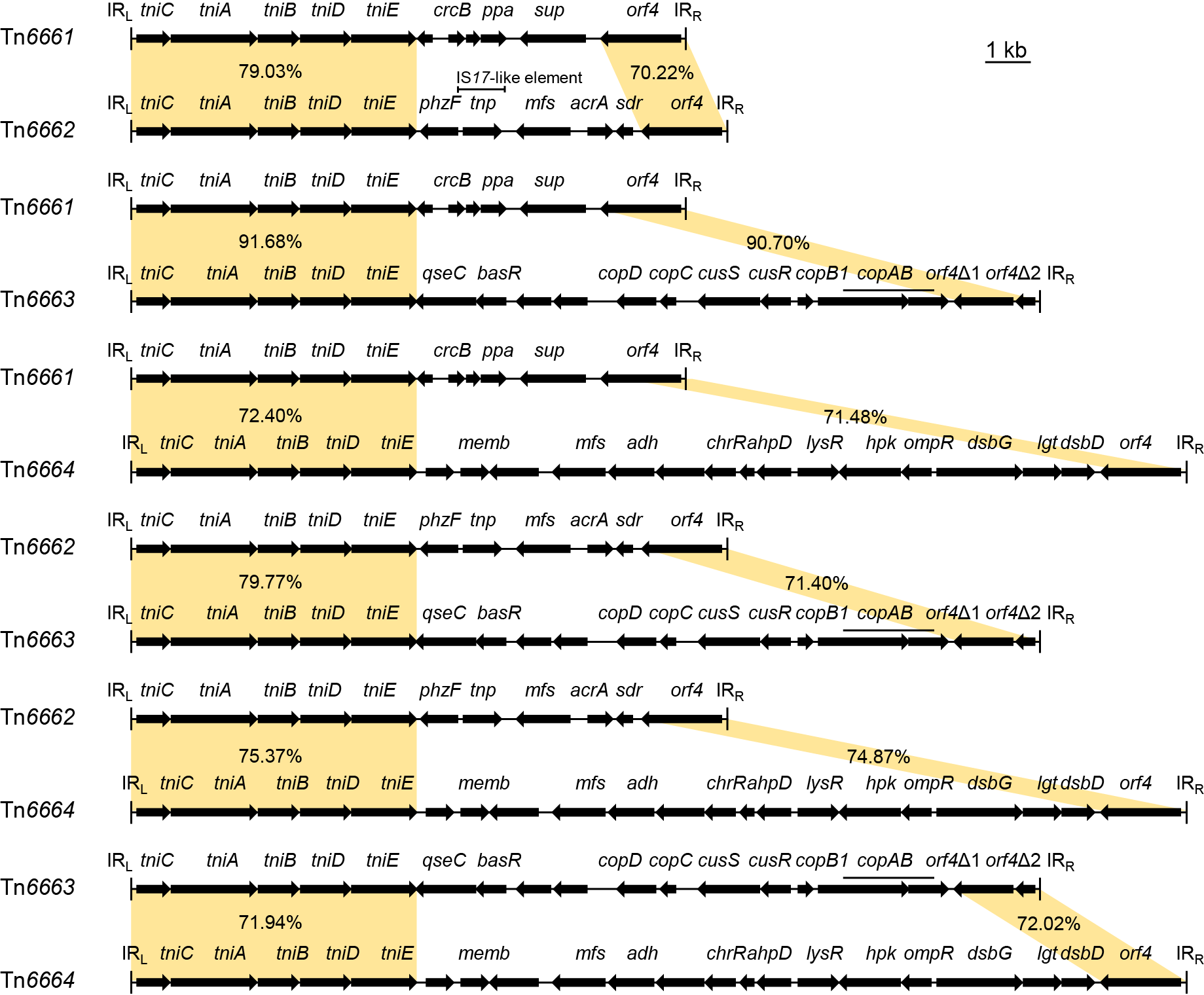

Supplement: FIG S3 [file mSphere.00349-20-sf003.tif]

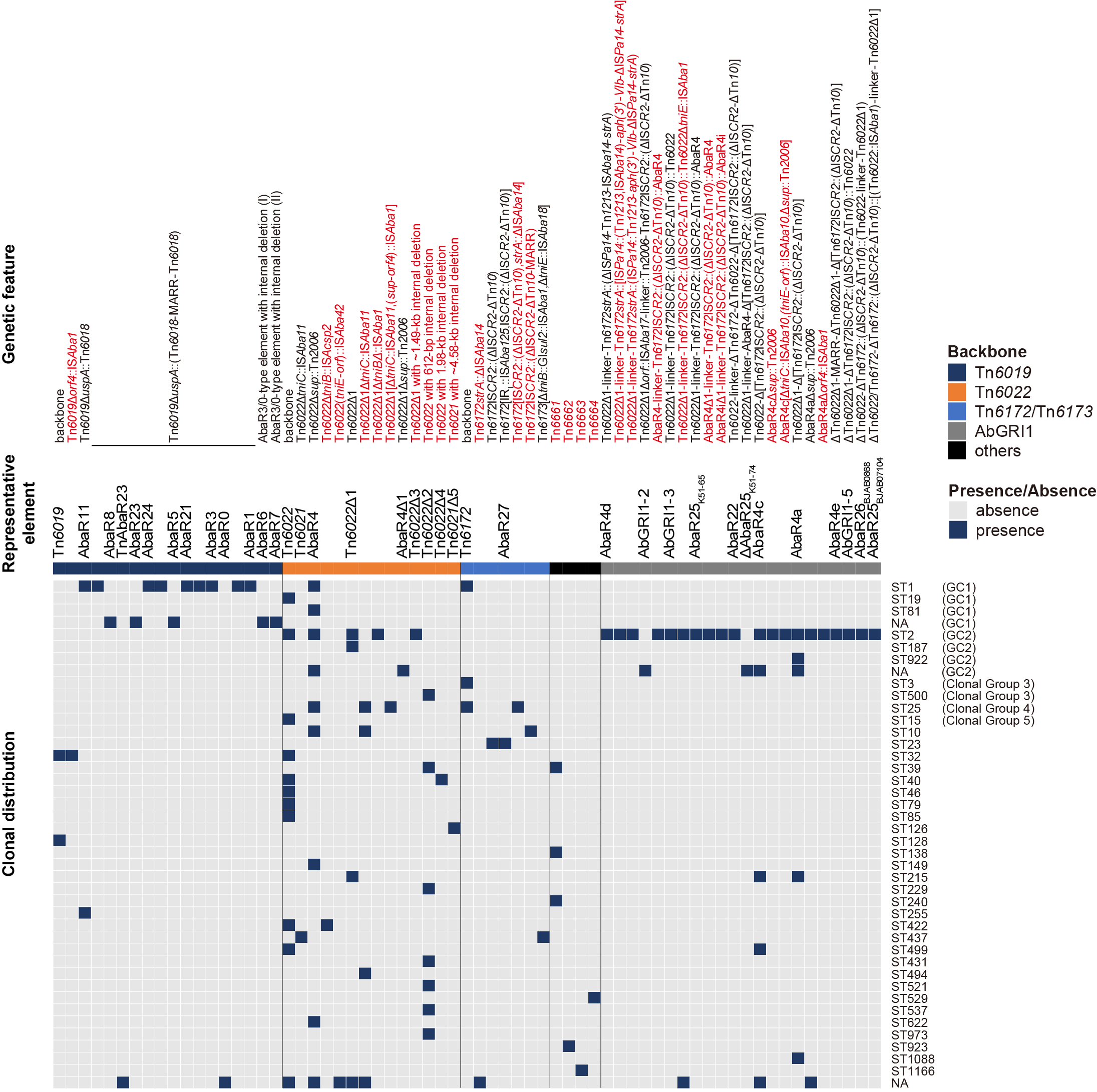

Supplement: FIG S4 [file mSphere.00349-20-sf004.tif]
