# Supplementary material for: Comparative Analysis of AbaR-Type Genomic Islands Reveals Distinct Patterns of Genetic Features in Elements with Different Backbones
Source: mSphere. 2020 May 27;5(3):e00349-20. doi: 10.1128/mSphere.00349-20 (PMC7253598; doi:10.1128/mSphere.00349-20)
Supplement: TABLE S2 [file mSphere.00349-20-st002.docx]

Table S2. Genetic configurations of the Tn*6022*- or Tn*6172*-derived variants that occurred only as part of an AbGRI1-type element.

| Backbone | Genetic configuration^a^ | Element | MGE(s) on the backbone | Antimicrobial resistance genes | Insertion site | Reference |
| --- | --- | --- | --- | --- | --- | --- |
| Tn*6022* | Tn*6022*Δ*tniE*::IS*Aba1* |  | IS*Aba1* |  | *tet*(B) | This study |
|  | Tn*6022*Δ*sup*::Tn*2006*(inverted) | AbaR4i^b^ | Tn*2006* | *bla*_OXA-23_ | *tet*(B) | This study |
|  | Tn*6022*[Δ*tniC*::IS*Aba10*,(*tniE*-*orf*)::IS*Aba10*,Δ*sup*::Tn*2006*]c |  | IS*Aba10*, Tn*2006* | *bla*_OXA-23_ |  | This study |
|  | Tn*6022*Δ1Δ*orf4*::IS*Aba1* |  | IS*Aba1* |  |  | This study |
|  | Tn*6022*Δ1Δ*sup*::Tn*2006*(inverted) | AbaR4iΔ1^b^ |  |  |  | This study |
|  | Tn*6022*Δ1Δ*orf*::IS*Aba17* | Part of Tn*6167*/AbGRI1-2 | IS*Aba17* |  |  | ([1](#_ENREF_1)) |
| Tn*6172* | Tn*6172strA*::(ΔIS*Pa14*-Tn*1213*-IS*Aba14*-*strA*) | Part of AbaR4d | ΔIS*Pa14*, Tn*1213*, IS*Aba14* | *sul2*, *strB*, *strA*, *bla*_PER-1_ |  | ([2](#_ENREF_2)) |
|  | Tn*6172strA*::[IS*Pa14*::(Tn*1213*,IS*Aba14*)-*aph(3')-VIb*-ΔIS*Pa14*-*strA*] |  | ΔIS*Pa14*, IS*Pa14*, Tn*1213*, IS*Aba14* | *sul2*, *strB*, *strA*, *bla*_PER-1_, *aph(3')-VIb* |  | This study |
|  | Tn*6172strA*::(IS*Pa14*::Tn*1213*-*aph(3')-VIb*-ΔIS*Pa14*-*strA*) |  | ΔIS*Pa14*, IS*Pa14*, Tn*1213* | *sul2*, *strB*, *strA*, *bla*_PER-1_, *aph(3')-VIb* |  | This study |

MGE, mobile genetic element

^a^ See Figs 4-5 for detailed schematic illustration.

^b^ Designated by this study. The letter "i" in the names "AbaR4iΔ1" and "AbaR4i" means the Tn*2006* in AbaR4iΔ1 and AbaR4i are inverted when comparing to that in AbaR4Δ1 and AbaR4, respectively.

**References for Table S2**

1. Nigro SJ, Hall RM. 2012. Tn*6167*, an antibiotic resistance island in an Australian carbapenem-resistant *Acinetobacter baumannii* GC2, ST92 isolate. J Antimicrob Chemother 67:1342-6.

2. Seputiene V, Povilonis J, Suziedeliene E. 2012. Novel variants of AbaR resistance islands with a common backbone in *Acinetobacter baumannii* isolates of European clone II. Antimicrob Agents Chemother 56:1969-73.
